# Measurable residual disease in multiple myeloma: ready for clinical practice?

**DOI:** 10.1186/s13045-020-00911-4

**Published:** 2020-06-22

**Authors:** Leire Burgos, Noemi Puig, Maria-Teresa Cedena, María-Victoria Mateos, Juan José Lahuerta, Bruno Paiva, Jesús F. San-Miguel

**Affiliations:** 1 Clínica Universidad de Navarra, Centro de Investigación Médica Aplicada (CIMA), Instituto de Investigación Sanitaria de Navarra (IDISNA), CIBER-ONC number CB16/12/00369, Pamplona, Spain; 2 Hospital Universitario de Salamanca, Instituto de Investigación Biomédica de Salamanca (IBSAL), Salamanca, Spain; 3 Hospital 12 de Octubre, CIBER-ONC number CB16/12/00369, Madrid, Spain

**Keywords:** Myeloma, Plasma cells, MRD, Clinical practice, Surrogate

## Abstract

The landscape of multiple myeloma (MM) has changed considerably in the past two decades regarding new treatments, insight into disease biology and innovation in the techniques available to assess measurable residual disease (MRD) as the most accurate method to evaluate treatment efficacy. The sensitivity and standardization achieved by these techniques together with unprecedented rates of complete remission (CR) induced by new regimens, raised enormous interest in MRD as a surrogate biomarker of patients’ outcome and endpoint in clinical trials. By contrast, there is reluctance and general lack of consensus on how to use MRD outside clinical trials. Here, we discuss critical aspects related with the implementation of MRD in clinical practice.

## Introduction

The outcome of patients with multiple myeloma (MM) has improved significantly in the last 20 years. This was the result of more than eight novel agents incorporated into the treatment armamentarium of MM, which led to unprecedented rates of complete remission (CR) and prolonged survival. In fact, we are now in a position to discuss whether MM may become a curable disease, which was beyond imagination a few years ago. Eradicating all tumor cells is a prerequisite to cure most malignancies, which raises the need of using high-sensitive tools to evaluate treatment efficacy. Although the definition of CR in MM is very useful in clinical practice, its sensitivity is suboptimal in many patients since current criteria relies on traditional techniques such as serum immunofixation and plasma cell (PC) enumeration by morphology that does not discriminate between normal and tumor cells. Adding immunohistochemistry or immunofluorescence does not improve cytological analysis and its sensitivity is low (10^−2^) due to the recovery of normal PCs after therapy that normalize kappa/lambda ratios. Furthermore, the serum free light-chain ratio has proven to be of limited value to discriminate patients in CR at different risk of progression and in fact, the stringent CR definition has failed to improve risk-stratification beyond conventional CR [1–3]. Therefore, the words “complete”–“remission” are misleading for many patients because they may interpret that, once achieved such status, the disease has been eradicated. Thus, it becomes evident that more sensitive techniques are needed to detect measurable (formerly called minimal) residual disease persisting below CR. Ideally, this would contribute to evaluate treatment efficacy with exquisite resolution (one that matches the high efficacy of new regimens) and to avoid both over and under treatment. Unfortunately, there is still a marked imbalance between the extraordinary therapeutic progress and the use of laboratory tests to monitor patients and, accordingly, to individualize treatments decisions in MM.

If response to therapy is one of the most, if not the most, effective marker to predict survival, who would not want to know with high precision, the quality of patients’ response to therapy? Should we ignore biological information with clear correlation with outcome?. We are now in 2020, but almost 20 years ago there was already evidence about the prognostic impact of persistent MRD in CR patients; should we wait for another two decades or should we implement this information to investigate innovative therapeutic interventions and to individualize patients’ management?

MRD techniques can be divided into those identifying extramedullary disease (e.g., positron emission tomography/computed tomography (PET/CT)) and those detecting intramedullary disease by either multiparameter flow cytometry (MFC) immunophenotyping or molecular assessment of immunoglobulin gene rearrangements. Using MFC, we can identify myelomatous PC based on light-chain clonality of phenotypically aberrant tumor cells. Initial MFC approaches (with a sensitivity of 10^−4^ and no standardization) [4, 5] have evolved into next-generation flow (NGF) cytometry developed by EuroFlow, which is based on optimized monoclonal antibodies combinations and sample preparation protocols that overcome blocking or internalization of monoclonal antibodies targeting PC antigens such as CD38, the acquisition of ≥ 10^7^ nucleated cells per sample, and novel software tools allowing for automated analysis with an expected sensitivity of 2 × 10^−6^ [6]. A similar evolution was observed on molecular grounds, where clonal immunoglobulin gene rearrangements (the unique ID of myelomatous PC) were initially identified by laborious and low-applicable ASO-PCR techniques and are now detected by next-generation sequencing (NGS) that performs millions of reads of DNA fragments in a standardized fashion with a sensitivity of 10^−6^ [7]. Both NGF and NGS have advantages and disadvantages for MRD detection that have been enumerated elsewhere [8, 9], but yield similar clinical results [10, 11] if used according to the guidelines of the International Myeloma Working Group (IMWG) [9]*.* NGS has been standardized through commercial kits developed by some companies and can be performed in frozen samples, which is an advantage for large multicenter clinical trials; NGF does not require baseline samples, allows evaluation of the whole bone marrow (BM) cellularity (e.g., hemodilution) and results are available in few hours. While both NGF and NGS supersede the performance of previous immunophenotypic and molecular methods, patients with undetectable MRD by any of these technologies continue to show a linear risk of relapse [12]. Thus, further improvement in the sensitivity of NGF and NGS are warranted to optimize risk-stratification based on patients’ MRD status. PET/CT is currently the optimal method to evaluate the disease outside the BM and there are ongoing efforts for its standardization [13]. Fluorodeoxyglucose is the most widely used radiolabeled compound but others such as methionine are under investigation [14]. PET/CT evaluation of treatment efficacy correlates with patients’ PFS [15–17]. Furthermore, studies from the IFM and University of Arkansas demonstrated complementarity between PET/CT and flow cytometry for risk-stratification [16, 18]. A recent analysis of PETHEMA/GEM uncovered that approximately half of patients with undetectable MRD developing early progression, some of them with extra-osseous plasmacytomas at diagnosis, presented new plasmacytomas as an isolated criterion of disease progression, without detectable M-protein or BM infiltration. Thus, it appears that these were true false-negative MRD results, reinforcing the need to combine NGF or NGS with PET/CT to monitor treatment efficacy, particularly in patients presenting with extramedullary or macro-focal disease, as well as elevated LDH levels [19].

Here, we will discuss critical aspects related with the implementation of MRD in clinical practice.

## Does undetectable MRD meet the key requirements to be used as treatment endpoint?

We considered the following prerequisites to evaluate if undetectable MRD can be used as treatment endpoint in MM: (1) must supersede the prognostic value of CR; (2) must provide reproducible results irrespectively of methodology and disease setting; and (3) must be applicable to all patients.

### MRD supersedes CR

Many studies have shown significant differences in progression-free (PFS) and overall survival (OS) between patients in CR with detectable vs undetectable MRD, and this was confirmed in a recent meta-analysis showing a hazard ratio (HR) of 0.44 (95% CI 0.34–0.56, *P* < .001) for PFS and of 0.47 (95% CI 0.33–0.67, *P* < .001) for OS in favor of those patients in CR who had undetectable MRD [20]. Another striking evidence that MRD supersedes CR is the study conducted by Lahuerta et al. [21] in a large MM series (797 cases). First, it was demonstrated that patients in CR have longer PFS and OS than those in very good partial response (VGPR)/near complete response (nCR), partial response (PR) or less than PR. However, upon discriminating patients in CR that were MRD negative and positive, it became evident that cases in CR with persistent MRD had the same outcome as patients in nCR/VGPR and even PR (PFS of 27 and 29 months, and OS of 59 and 65 months, respectively). These results underpin that the true value of CR is intimately connected to the subset of patients in CR that have undetectable MRD: the higher the frequency of undetectable MRD the better the outcome of CR patients [21].

### The clinical impact of MRD is reproducible in different centers, by molecular and immunophenotypic methods, and in all disease settings

Recent studies in the transplant setting have reported groundbreaking results using NGS and NGF [19, 22]. With a sensitivity in the logarithmic range of 10^−6^, both provided similar and dramatic discrimination between patients with undetectable vs persistent MRD, which resulted in HR for PFS of 0.22 (95% CI 0.15–0.34; *P* < .001) with NGS and 0.18 (95% CI 0.11–0.30; *P* < .001) with NGF. This confirms that both techniques are equally robust for risk-stratification and illustrates the reproducibility between different centers/groups regarding clinical outcomes according to MRD results. Indeed, a subanalysis of the CASSIOPEIA study conducted by the French group that compares both techniques at the sensitivity level of 10^−5^, showed high correlation [10]. Large studies such as the UK Myeloma XI [23] and the EMNO2/MO95 [24] conducted by other centers/groups that used MFC with a sensitivity ranging from 10^−4^ to 10^−5^, were able to reproduce the prognostic impact of MRD (HR for PFS of 0.19 and 0.44, respectively).

Until recently, information in transplant-ineligible patients was less abundant probably because achieving CR was infrequent in this setting. Two large randomized trials comparing VMP or Rd with or without daratumumab (ALCYONE and MAIA, respectively) demonstrated that independently of treatment, those patients achieving undetectable MRD by NGS enjoyed significantly longer PFS [25, 26]. Similar results were described in the CLARION trial using NGF [27]. Of note, the Spanish group has shown that the impact of MRD negativity in reducing the risk of progression and/or death is higher in the elderly as compared to transplant-eligible patients [21]. We believe this reflects the impact of initial depth of response in a patient population with limited options to receive more than 2–3 lines of therapy due to age and comorbidities [28]. New options for salvage therapy have markedly increased depth of response and survival in patients with relapsed/refractory MM. In this setting, the most solid MRD information derives from two randomized studies using NGS: CASTOR (bortezomib/dexamethasone ± daratumumab) [29] and POLLUX (lenalidomide/ dexamethasone ± daratumumab) [30]. Both confirmed that irrespective of treatment, MRD-negative patients had significantly longer PFS. Altogether, these results confirm that the clinical value of reaching MRD negativity is independent of the treatment received, which has been reproduced in different studies by different groups using different techniques. This is supported by the meta-analysis of Munshi et al. [20].

### Undetectable MRD is clinically relevant in patients with standard- and high-risk disease

It is well-stablished that MM patients with high-risk cytogenetics have poor outcome. While the achievement of CR commonly fails to prolong survival in this population, the Spanish group showed that the impact of achieving MRD negativity in reducing the risk of progression and/or death is even higher in patients with adverse cytogenetics than in standard-risk cases [19, 21]. The French group has confirmed that MRD status by NGS not only discriminates outcomes in both standard- and high-risk patients, but also that if the later population achieve an undetectable MRD their PFS will be longer than those with standard-risk cytogenetics but persistent MRD [22]. Similar results have been reported by NGF [19]; the median PFS was similar for MRD-negative patients with revised International staging system (R-ISS) 1, 2, and 3 (not reached in any category), while in the MRD-positive population the median PFS was not reached for R-ISS 1, and it is 38 months and 14 months for R-ISS 2 and 3, respectively. These results reinforce the predictive value of MRD in standard and high-risk MM and unveil that risk is dynamic, since patients with adverse prognosis may shift into favorable once upon achieving deep responses to treatment with undetectable residual tumor cells [19]. These findings suggest that the only way to overcome the dismal outcome of high-risk patients is by considering undetectable MRD as their treatment endpoint.

## Potential pitfalls of MRD in MM

All the above suggests that MRD meets the key requirements to become a treatment endpoint in MM. However, the potential pitfalls of MRD techniques should be recognized and have been summarized below in four items:

### The quality of BM samples

MM displays a patchy pattern of BM infiltration and, irrespectively of that, samples can be hemodiluted. Accordingly, we cannot be totally certain that an MRD-negative result, irrespectively of the technique used, represents real absence of clonal PC or is due to sampling error. To minimize a false MRD-negative result, the presence of BM cellular elements should be evaluated [6] and an MRD-negative result should be confirmed in a second (or more) assessment [9].

### Patients displaying transient or unsustained MRD negativity

In line with what is required for definition of CR (a confirmatory sample), for MRD it has become evident that although MRD negativity in a single time point clearly predicts longer survival, risk-stratification is significantly improved when this result is reproduced at 6 or 12 months. (POLLUX and CASTOR studies [31]).

### Cases remaining MRD positive at very low levels without disease progression

This may be explained by the presence of “benign” MRD clones and/or a very active immune reconstitution with the capacity to control low numbers of residual clonal cells [28]. The Spanish group and others have shown a few patients with unique immune features may have prolonged PFS despite persistent MRD [28, 32].

### Persistence of extramedullary disease not detectable in BM aspirates

The extended use of PET/CT in MM assessment has illustrated that not only extramedullary, but also paramedullary and single focal lesions can be present and undetectable with conventional MM exams [33]. In fact, there is now consensus that evaluation, both inside and outside the BM, is the best option to detect residual disease, and patients that are double negative for these two complementary assessments have the best outcome [9, 18].

## How to implement MRD in clinical practice?

Since MRD is one of the most (if not the most) relevant prognostic factor, we should take advantage of this information to improve patient management (including both for innovative clinical trial—e.g., Table 1—design and in clinical practice). Naturally, MRD assessment should be performed only when a BM aspirate is collected to confirm CR, in accordance to the IMWG guidelines [9]. From thereafter, MRD testing should be performed whenever such results could help on clinical decisions (e.g., in between treatment stages) and repeated periodically (e.g., every 1 or 2 years) to confirm patients’ MRD status. First, it is important to clarify that while, as discussed above, an MRD-negative result still has a certain degree of uncertainty, persistence of MRD is a strong adverse prognostic feature, even among CR patients. Accordingly, it will be safer to make clinical decisions based on persistent MRD than on undetectable MRD.

**Table 1:** Clinical trials where MRD guides treatment decisions. Results are based on a search in the ‘https://clinicaltrials.gov/’ website that included the terms “multiple myeloma” and “MRD”, and individual identification of clinical trials where treatment algorithms were triggered by patients’ MRD status. Selected studies (identified with an asterisk) were added based on knowledge of their existence, despite being absent in search results. It should be noted that many more studies assess MRD and in most clinical trials, MRD response rates are a primary or secondary endpoint (see Fig. 1). However, because no apparent treatment decision is being made based on patients’ MRD status, those studies were not included in the table below. There are many clinical trials that, to the best of our knowledge, will have MRD-guided treatment decisions but were not added because their design is still being finalized or have not been initiated at the time of this publication

NCT	Study official title	Country	Technique
NCT02406144	Maintenance treatment with lenalidomide and dexamethasone versus lenalidomide, dexamethasone and ixazomib after autologous hematopoietic stem cell transplantation in patients With newly diagnosed symptomatic multiple myeloma-duration of maintenance guided by MRD status (GEM2014MAIN)	Spain	NGF
RADAR*	Risk adapted therapy directed according to response comparing treatment escalation and de-escalation strategies in newly diagnosed patients with multiple myeloma suitable for stem cell transplantation	UK	N/A
NCT03490344	Short course daratumumab in minimal residual disease (MRD) positive myeloma patients after induction therapy with/without consolidative high-dose chemotherapy/autologous stem cell support	USA	FC
NCT03224507	Monoclonal antibody-based sequential therapy for deep remission in multiple myeloma (MASTER)	USA	NGS
NCT03742297*	Induction therapy with bortezomib-melphalan and prednisone (VMP) followed by lenalidomide and dexamethasone (Rd) versus carfilzomib, lenalidomide, and dexamethasone (KRd) plus/minus daratumumab, 18 cycles, followed by consolidation and maintenance therapy with lenalidomide and daratumumab: phase III, multicenter, randomized trial for elderly fit newly diagnosed multiple myeloma patients aged between 65 and 80 years	Spain	NGF
NCT03697655	Pre-emptive daratumumab therapy of minimal residual disease reappearance or biochemical relapse in multiple myeloma (PREDATOR)	Poland	N/A
NCT03710603	A phase 3 study comparing daratumumab, VELCADE (Bortezomib), lenalidomide, and dexamethasone (D-VRd) vs VELCADE, lenalidomide, and dexamethasone (VRd) in subjects with previously untreated multiple myeloma who are eligible for high-dose therapy (PERSEUS)	EMN	N/A
NCT03992170	A pilot study on the efficacy of daratumumab in multiple myeloma (MM) patients in >VGPR/MRD-positive by next-generation flow (DART4MM)	Italy	FC
NCT02969837	Open-label, single-arm, phase 2 study of initial treatment with elotuzumab, carfilzomib (Kyprolis), lenalidomide (Revlimid), and low-dose dexamethasone (E-KRd) in newly diagnosed, multiple myeloma requiring systemic chemotherapy	USA	NGS and MFC
NCT04071457	S1803, phase III study of daratumumab/rHuPH20 (NSC-810307) + lenalidomide or lenalidomide as post-autologous stem cell transplant maintenance therapy in patients with multiple myeloma (MM) using minimal residual disease to direct therapy duration (DRAMMATIC study)	USA	NGS
NCT04096066	Carfilzomib-lenalidomide-dexamethasone (KRd) versus lenalidomide-dexamethasone (Rd) in newly diagnosed myeloma patients not eligible for autologous stem cell transplantation: a randomized phase III trial	Italy	N/A
NCT03376477	A randomized, double-blind, placebo-controlled phase II trial of an allogeneic myeloma GM-CSF vaccine with lenalidomide in multiple myeloma patients in complete or near complete	USA	NGS
NCT04108624	A multimodality approach to minimal residual disease detection to guide post-transplant maintenance therapy in multiple myeloma (MRD2STOP)	USA	NGS
NCT04221178	A single-arm, prospective atudy of maintenance therapy cessation for patients with multiple myeloma in sustained MRD-negative remissions	USA	NGF
NCT04140162	Phase 2 study with minimal residual disease (MRD) driven adaptive strategy in treatment for newly diagnosed multiple myeloma (MM) with upfront daratumumab-based therapy	USA	N/A

*NGS* next-generation sequencing, *FC* flow cytometry, *NGF* next-generation flow cytometry, *N/A* not available, *EMN* European Myeloma Network

**Fig. 1:**
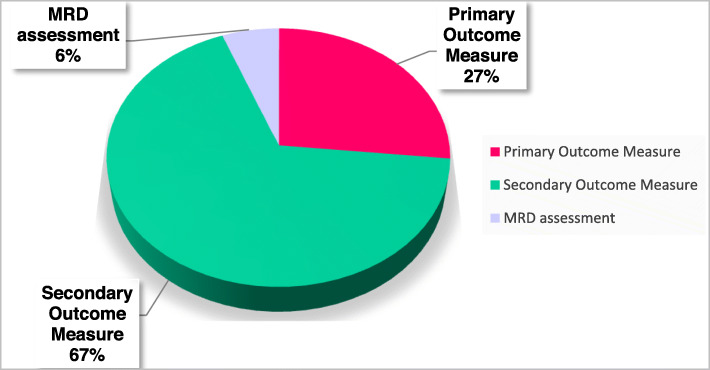
Clinical trials reporting MRD assessment. Results are based on a search in the https://clinicaltrials.gov/ website that included the terms “multiple myeloma” and “MRD.” Of 170 clinical trials, 154 indicate in the “descriptive information” that MRD is assessed. Furthermore, MRD negative rates are a “primary outcome measure” in 41 (27%) studies, and a “secondary outcome measure” in 104 (67%) trials

How to take advantage of the higher sensitivity of modern MRD techniques to evaluate treatment efficacy and to guide therapeutic decisions? Table 1 shows the ongoing clinical trials that use MRD assessment using next-generation techniques. As a first example, if a patient is in CR before ASCT, how to evaluate the efficacy of subsequent high-dose therapy? In this context, you may be guided by the effect of high-dose therapy on persistent MRD. Similarly, if the patient is reluctant or is not a candidate for ASCT and has achieved CR, why not continue with additional cycles of consolidation until MRD becomes undetectable before moving to maintenance? Second, in high-risk patients with persistent MRD following an optimized induction plus ASCT, we know median PFS will be very short (typically less than two years) [21, 22]; accordingly, it could be envisioned that the introduction of novel agents such as monoclonal antibodies plus second/third generation of PI/IMIDS after ASCT may produce benefit; noteworthy, this “risk-adapted therapy approach” is being tested in some trials. Third, if we know that treatment “A” induces three-fold higher MRD-negative rates as compared to treatment “B,” should this influence my clinical practice? Fourth, to adapt maintenance intensity and duration. Several clinical trials are investigating this concept; for example, the RADAR study from the UK group segregates MRD-positive and MRD-negative patients after ASCT: in the first cohort, they will compare 1 vs 2 vs 3 drugs (IMID-PI-MoAb), while in MRD-negative cases, they will explore treatment until disease progression versus fixed duration. Similarly, in the Spanish GEM2014MAIN trial, after 2 years maintenance patients were randomized according to MRD: if positive, they continued for 3 years but if negative, they stopped treatment [19]. These are selected examples out of many other trials with similar conceptual design, all oriented to stop maintenance if the patient is MRD-negative and continue if positive. However, it can be argued that if patients remain MRD-positive after optimal intensive treatment (including 3–4 drugs), maintenance with a single agent will be of limited value and probably, these cases may benefit from an experimental approach (e.g., individualized immunotherapy according to tumor and immune cell biology). By contrast, if patients have undetectable MRD, standard maintenance approaches may effectively maintain immune surveillance and sustain undetectable MRD for long periods of time. Accordingly, data from most recent studies suggest that patients with undetectable MRD are the ones that (as opposed to cases with persistent MRD) benefit the most from maintenance therapy [19, 22, 25, 26]. We believe this is the surrogate biomarker of cure in MM, and trials designed to address these concepts are of utmost importance (Table 1). In fact, the notion that MRD can act as surrogate biomarker for survival and thereby accelerate drug development is evolving based on consistent and positive results observed in recent years (Table 2), and a progressive number of clinical trials are using MRD rates as primary endpoint (Fig. 1).

**Table 2:** Prospective randomized clinical trials with MRD assessment using next-generation techniques. These studies were selected based on reported effect of treatment randomization in patients’ outcome and MRD negativity rates. Overall, whenever significant differences in MRD rates are observed, these predicted significant differences in outcome

Study	Treatment	Outcome	MRD assessment	MRD-negative rate
*Transplant-eligible*				
IFM2009 (NCT01191060) [22, 34]	HDT vs RVD	HDT: median PFS 50m RVD: median PFS 36m	NGS (10^−6^)^a^	HDT 30% RVD 20%
CASSIOPEIA (NCT02541383) [10]	D-VTd vs VTd	D-VTD: 18m PFS 93% VTD: 18m PFS 85%	NGF (10^−5^)	D-VTD 64% VTD 44%
*Transplant-ineligible*				
ALCYONE (NCT02195479) [25]	D-VMP vs VMP	D-VMP: median PFS NR VMP: median PFS 18.1m	NGS (10^−5^)	D-VMP 22.3% VMP 6.2%
CLARION (NCT01818752) [27]	KMP vs VMP	KMP: median PFS 22.3m VMP: median PFS 22.1m	NGF (10^−6^)	KMP 15.7% VMP 15.5%
MAIA (NCT02252172) [26]	DRd vs Rd	DRd: median PFS NR Rd: median PFS 31.9m	NGS (10^−5^)	DRd 24.2% Rd 7.3%
*Relapse/refractory*				
POLLUX (NCT02076009) [30]	DRd vs Rd	DRd: median PFS NR Rd: median PFS 17.5m	NGS (10^−5^)	DRd 22.4% Rd 4.6%
CASTOR (NCT02136134) [29]	DVd vs Vd	DVd: median PFS 16.7m Vd: median PFS 7.1m	NGS (10^−5^)	DVd 11.6% Vd 2.4%

*MRD* measurable residual disease, *HDT* high-dose therapy, *D* Daratumumab, *V* Bortezomib, *T* Thalidomide, *d* Dexamethasone, *M* Melphalan, *P* Prednisone, *K* Carfilzomib, *R* Lenalidomide, *PFS* progression-free survival, *m* months, *NR* not reached, *NGF* next-generation flow, *NGS* next-generation sequencing

^a^This study also reported MRD rates based on a 7-color flow cytometry assay that, similarly to the results obtained by NGS, showed significant differences between the HDT vs RVD arm.

## Looking into the future

The longer an undetectable MRD status is sustained, the higher its impact in reducing the risk of progression and prolonging survival of MM patients. This requires periodic MRD assessment and invasive BM aspirates pose a challenge. Thus, further methodological innovation is warranted to monitor MRD in blood as frequent as possible. Of note, promising results have been recently reported using NGS [35] and NGF [36] in peripheral blood (PB). Namely, the EuroFlow consortium as recently reported that with NGF, it was possible to detect MRD in PB of 17% of patients in CR; most importantly, presence of MRD in PB identified a subgroup of patients in CR with dismal outcome (median PFS of 9 months) [37]. Conversely, both studies using NGS and NGF showed that approximately 40% of patients displayed MRD in BM that was undetectable in PB [35, 36]. There is also growing evidence supporting circulating tumor DNA (ctDNA) for liquid biopsies in MM. However, this approach suffers from a conundrum between applicability and extent of genetic information: while targeted sequence of a few genes or hotspot mutations is highly applicable, comprehensive whole-exome sequencing of cfDNA is possible in a small number of patients with high ctDNA burden [38–40]. Thus, these approaches do not seem to be powered for sensitive MRD assessment in all MM patients. By contrast, matrix-assisted laser desorption ionisation time-of-flight mass spectrometry that detect M-proteins in serum has shown to be more sensitive compared to current electrophoretic methods [41, 42]. In fact, most recent observations suggest that this method may provide complementary information to MRD assessment in BM [43, 44]. More studies are needed to define if this concept is ready for prime time. We believe that a minimally-invasive MRD test will foster its use in clinical practice, particularly for preemptive therapeutic approaches upon MRD reappearance in PB. However, at the moment, we consider that BM remains the gold-standard sample for MRD testing.

We must not forget that under the pressure of enormous drug costs, the best way to make our health systems sustainable is by curing MM. We have experienced great progress and now we need to optimize the use of highly effective drugs developed including emerging immunotherapeuties. This should be implemented early in the course of the disease in order to overcome the poor prognosis of high-risk patients, including those with persistent MRD after optimal frontline treatment. In other words, “early detection of the problem guided by sensitive methods to allow early intervention.”

## Supplementary information

**Additional file 1.** List of investigators in the GEM (Grupo Español de Mieloma)/PETHEMA (Programa para el Estudio de la Terapéutica en Hemopatías Malignas) cooperative study group.

## Data Availability

Not applicable

## Abbreviations

MM: Multiple myeloma
MRD: Measurable residual disease
CR: Complete remission
PC: Plasma cell
PET/CT: Positron emission tomography/computed tomography
MFC: Multiparameter flow cytometry
NGF: Next-generation flow
NGS: Next-generation sequencing
IMWG: International Myeloma Working Group
BM: Bone marrow
PFS: Progression-free survival
OS: Overall survival
HR: Hazard ratio
VGPR: Very good partial response
nCR: Near complete response
PR: Partial response
R-ISS: Revised International staging system
PB: Peripheral blood
ctDNA: Circulating tumor DNA
