# Supplementary material for: Measurable residual disease in multiple myeloma: ready for clinical practice?
Source: J Hematol Oncol. 2020 Jun 22;13:82. doi: 10.1186/s13045-020-00911-4 (PMC7310444; doi:10.1186/s13045-020-00911-4)
Supplement: Supplementary file 1 — Additional file 1. List of investigators in the GEM (Grupo Español de Mieloma)/PETHEMA (Programa para el Estudio de la Terapéutica en Hemopatías Malignas) cooperative study group. [file 13045_2020_911_MOESM1_ESM.docx]

**List of investigators in the GEM (Grupo Español de Mieloma)/PETHEMA (Programa para el Estudio de la Terapéutica en Hemopatías Malignas) cooperative study group.**

| **Hospital** | **Investigator** |
| --- | --- |
| Complejo Hospitalario Costa del Sol | Dra. María Casanova Espinosa |
| H. Especialidades de Jerez de la Frontera | Dr. José Luís Guzman Zamudio |
| H. Nuestra Señora de Valme | Dr. Eduardo Ríos Herranz |
| H. Universitario Virgen de las Nieves | Dr. Rafael Rios Tamayo |
| Complejo Hospitalario Regional Virgen del Rocío | Dr. Jesús Martín Sánchez |
| H. Clínico Universitario Lozano Blesa | Dr. Luís Palomera Bernal |
| H. Universitario Central de Asturias | Dra. Ana Pilar González Rodríguez |
| H. Cabueñes | Dra. María Esther González García |
| Complejo Asistencial Son Espases | Dra. Antonia Sampol Mayol |
| H. Son Llátzer | Dr. Joan Bargay Lleonart |
| H. de Gran Canaria Dr. Negrín | Dra. Alexia Suárez |
| H. Universitario de Canarias | Dr. Miguel Teodoro Hernández García |
| H. Universitario Marqués de Valdecilla | Dra. Carmen Montes Gaisán |
| H. General de Ciudad Real | Dra. Belén Hernández Ruiz |
| Complejo Hospitalario de Toledo | Dr. Felipe Casado Montero |
| H. Universitario de Guadalajara | Dra. Dunia de Miguel Llorente |
| H. Nuestra Señora del Prado (Talavera) | Dr. Fernando Solano Ramos |
| H. General de Albacete | Dra. Ángela Ibañez Garcia |
| H. Clínico de Salamanca | Dra. Mariví Mateos Manteca |
| Complejo Hospitalario H. General de Segovia | Dr. José Mariano Hernández Martín |
| H. de León | Dr. Fernando Escalante Barrigón |
| H. Universitario Rio Hortega | Dr. Javier García Frade |
| H. Clínico Universitario de Valladolid | Dr. Alfonso García de Coca |
| H. Santa Bárbara | Dr. Carlos Aguilar Franco |
| Hospital Universitario de Burgos | Dr. Jorge Labrador Gómez: |
| H. Althaia, Xarxa Asistencial de Manresa (Sant Joan de Deu) | Dra. Elena Cabezudo Pérez |
| H CLINIC | Dr. Joan Bladé Creixentí |
| H. Durán i Reynals - ICO L´Hospitalet | Dra. Ana Mª Sureda Balari |
| ICO Girona, H. Universitario de Girona Dr. Josep Trueta | Dra. Yolanda González Montes |
| H. UNIVERSITARI JOAN XXII DE TARRAGONA | Dra. Lourdes Escoda Teigell |
| Hospital Universitari Arnau de Vilanova de Lleida | Dr. Antonio García Guiñón |
| H. del Mar | Dra. Eugenia Abella Monreal |
| H. de Sabadell (Parc Taulí) | Dr. Juan Alfonso Soler Campos |
| Hospital Universitario Mútua de Terrassa | Dr. Josep Mª Martí Tutusaus |
| H. Germans Trias i Pujol | Dr. Albert Oriol Rocafiguera |
| H. de la Santa Creu i Sant Pau | Dr. Miquel Granell Gorrochategui |
| H. Vall d´Hebrón | Dra. Mercedes Gironella Mesa |
| H. San Pedro de Alcántara (Complejo Hospitalario de Cáceres) | Dra. Carmen Cabrera Silva |
| Complejo Hospitalario Universitario de Santiago | Dra. Marta Sonia González Pérez |
| Complejo Hospitalario de Pontevedra | Dra. Ana Dios Loureiro |
| Complejo Hospitalario de Ourense | José Angel Méndez Sánchez |
| H. San Pedro | Dra. María Josefa Nájera Irazu |
| H. Universitario Fundación de Alcorcón | Dr. Francisco Javier Peñalver Párraga |
| H. Universitario 12 de Octubre | Dr. Juan José Lahuerta Palacios |
| H. de Fuenlabrada | Dra. Pilar Bravo Barahona |
| H. General Universitario Gregorio Marañón | Dra. Cristina Encinas Rodríguez |
| H. Infanta Leonor | Dr. José Ángel Hernández Rivas |
| H. Universitario Madrid - Sanchinarro | Dr. Jaime Pérez de Oteyza |
| Centro Oncológico MD Anderson | Dra. Rebeca Iglesias del Barrio |
| H. Universitario La Paz | Dra. Ana López de la Guia |
| H. Universitario de la Princesa | Dr. Adrián Alegre Amor |
| Fundación Jiménez Díaz-UTE | Dra. Elena Prieto Pareja |
| Hospital Universitario Puerta de Hierro - Majadahonda | Dra. Isabel Krsnik Castelló |
| H. Ramón y Cajal | Dra. Mª Jesús Blanchard Rodríguez |
| H. Universitario de San Carlos | Dr. Rafael Martínez Martínez |
| H. Severo Ochoa | Dra. Rosalía Riaza Grau |
| H. INFANTA SOFÍA | Dr. Eugenio Giménez Mesa |
| HOSPITAL DEL TAJO | Dra. Elena Ruiz Sainz |
| H. Morales Meseguer | Dr. Felipe de Arriba |
| H. Universitario Virgen de la Arrixaca | Dr. Jose María Moraleda Jiménez |
| H. General Universitario Santa Lucia | Dra. Marta Romera |
| Clínica Universidad de Navarra | Dr. Felipe Prósper Cardoso |
| Complejo Hospitalario de Navarra | Dr. José Mª Arguiñano Pérez |
| H. de Cruces | Dra. María Puente Pomposo |
| H. de Txagorritxu | Dr. Ernesto Pérez Persona |
| H. Clínico Universitario de Valencia | Dra. Ana Isabel Teruel Casasús |
| H. Universitario Dr. Peset | Dra. Paz Ribas García |
| H. Universitario La Fe | Dr. Isidro Jarque Ramos |
| H. General Universitario de Alicante | Dra. María Blanca Villarrubia Lor |
| H. TORREVIEJA SALUD UTE | Dr. Pedro Luis Fernández García |
| H. del Vinalopo | Dr. Pedro Luis Fernández García |
| H. Quirón | Dra. Carmen Martínez Chamorro |
